# New node on the block for organic solid-state chemists: *rtct*-tetra­kis­(pyridin-4-yl)cyclo­butane

**DOI:** 10.1107/S2053229621002205

**Published:** 2021-02-25

**Authors:** Eric W. Reinheimer

**Affiliations:** a Rigaku Americas Corporation, 9009 New Trails Drive, The Woodlands, TX 77381, USA

**Keywords:** crystal engineering, hydrogen bonding, [2+2] cyclo­addition reaction, organic solid state, inter­penetration, resorcinol, photoproduct, topochemical postulate

## Abstract

We now know the symmetry and unit-cell parameters of the functionalized cyclo­butane ***rtct*** **-TPCB**, as well as a multicomponent solid containing it. What other networks and topologies are possible?

As chemists and crystallographers, growing a crystal and obtaining the structure of a synthesized mol­ecule is often considered the dénouement of a sometimes long and often arduous process. To peer into the atomic world and view the three-dimensional model of a mol­ecule that might determine future research projects is the greatest reward for these inter­minable hours in the lab. While the mol­ecules and the conclusions may outlast your graduate, postdoctoral, or professional career, are all syntheses and applications truly complete with that crystal? Is that the end or can more be done?

Seminal work by Schmidt and co-workers in 1971 signaled the advent of *organic solid-state syntheses* and demonstrated that even within a single crystal additional synthetic work was possible. In their report, Schmidt reported that *trans*-cinnamic acid and its derivatives were capable of undergoing a [2 + 2] photodimerization to generate functionalized cyclo­butanes *post*-crystallization (Schmidt, 1971 ▸). As a direct result of this observation, the authors ultimately proposed the ‘Topochemical Postulate’, which hypothesized that olefin-containing mol­ecules within 4.2 Å of one another can undergo a photodimerization in the solid state.

While cinnamic acid crystallizes in such a way that the olefin is rendered photoactive, a far greater number of olefin-containing mol­ecules as a consequence of their crystal packing are photostable and are unable to undergo a photodimerization reaction. Pioneering research in crystal engineering has demonstrated that in order to perpetuate the onset of photoreactivity in these latter instances, the use of templates is required to align the reactant mol­ecules in order to photoreact (Gan *et al.*, 2018 ▸). Numerous supra­molecular inter­actions, such as hydrogen and halogen bonding, argentophilic and aurophilic inter­actions, along with others, have proven to be reliable in the formation of reactive solids containing olefins that are photostable as a single-component solid. This templated approach to solid-state synthesis has yielded novel mol­ecules (*i.e.* cyclo­phanes and ladderanes) whose yield is prohibitively low when prepared through a standard solution-based approach. The progress of the solid-state syntheses can be followed by ^1^H NMR as the disappearance of olefinic H atoms coupled with the concomitant appearance of H atoms from cyclo­butane moieties signals the onset of photoproduct formation (Campillo-Alvarado *et al.*, 2019 ▸; Chanthapally *et al.*, 2014 ▸; MacGillivray *et al.*, 2000 ▸; Pahari *et al.*, 2019 ▸; Sezer *et al.*, 2017 ▸; Volodin *et al.*, 2018 ▸).

Even though the solid-state syntheses of functionalized cyclo­butanes have yielded some very aesthetically appealing mol­ecules and crystal structures, further synthetic efforts utilizing these systems can be undertaken with the rational design of highly-connected networks. Careful consideration of both the nodes and linkers is essential given their direct influence on the topology of the network (Jiang *et al.*, 2018 ▸). Functionalized cyclo­butane mol­ecules generated using the [2 + 2] photodimerization reaction have been successfully incorporated as nodes within these connected networks. A survey of the literature has demonstrated that the most studied photoproduct within these networks is the *rctt*-isomer of tetra­kis­(pyridin-4-yl)cyclo­butane (***rctt*** **-TPCB**), which has functioned as a node in both metal-based and mol­ecular networks (Li *et al.*, 2014 ▸; Blake *et al.*, 1997 ▸; Oburn *et al.*, 2020 ▸; Baldrighi *et al.*, 2010 ▸). While the *rctt*-isomer of tetra­kis­(pyridin-4-yl)cyclo­butane is the most-often utilized, a second isomer (***rtct*** **-TPCB**) is known and considered to be more thermodynamically stable. The H_2_SO_4_-catalyzed procedure to prepare the *rtct*-isomer from its *rctt* homologue in dimethyl sulfoxide (DMSO) was first outlined in 2010 (Peedikakkal *et al.*, 2010 ▸); however, no single-crystal structure of ***rtct*** **-TPCB** or a structure demonstrating that it could, like ***rctt-*** **TPCB**, be incorporated into a purely mol­ecular network was reported.

Thanks to the recent report by the Groeneman research group, the lack of a structure for the ***rtct*** **-TPCB** isomer (Fig. 1 ▸), as well as structural proof that it can function as a node in a mol­ecular network, has been clarified for the solid-state and crystal engineering communities (Santana *et al.*, 2021 ▸). Within the extended structure, ***rtct*** **-TPCB** functions as a three-con­necting node by accepting three O—H⋯N hydrogen bonds with included water mol­ecules and 4,6-di­chloro­resorcinol (Fig. 2 ▸). This type of packing arrangement for the node is required to form the ornate honeycomb topology (Fig. 3 ▸). Ultimately, it was found that the honeycomb network formed a three-dimensional network through the onset of C—H⋯N hydrogen bonding between a H atom on the cyclo­butane and the final pyridyl N atom on a neighboring ***rtct*** **-TPCB**.

Due to this highlighted work, we now know the symmetry and unit-cell parameters of the functionalized cyclo­butane ***rtct*** **-TPCB**, as well as a multicomponent solid containing it. What other networks and topologies are possible? With the facile, acid-catalyzed procedure to generate other regio­isomers, it is exciting to envision additional regioisomers of other functionalized cyclo­butanes yet to be reported. The authors are to be applauded for finally addressing the paucity of information on this under-utilized isomer and shedding some light on it.

## Figures and Tables

**Figure 1:**
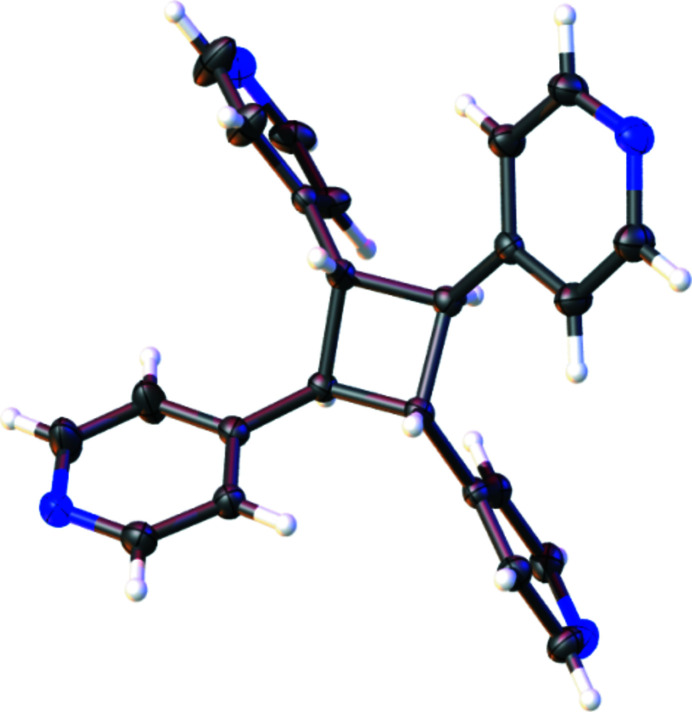
Rendering of the asymmetric unit of ***rtct*** **-TPCB**. Anisotropic displacement ellipsoids have been set at the 50% probability level.

**Figure 2:**
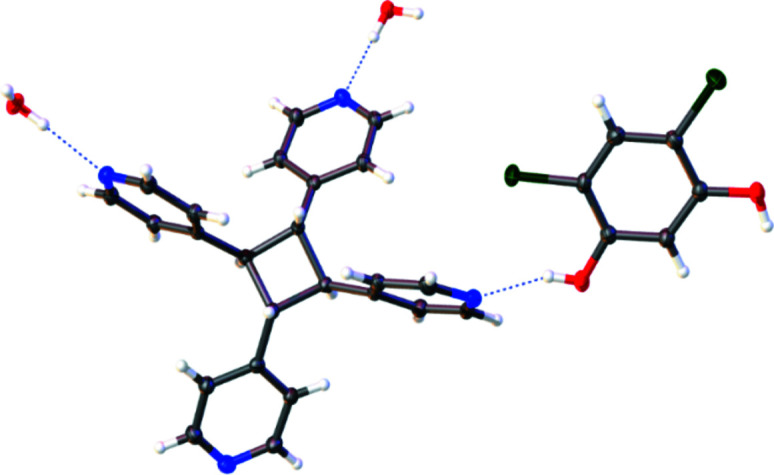
The functionalized cyclo­butane ***rtct*** **-TPCB** functioning as a hydrogen-bond-accepting three-connecting node by engaging in O—H⋯N hydrogen bonding. Anisotropic displacement ellipsoids have been set at the 50% probability level.

**Figure 3:**
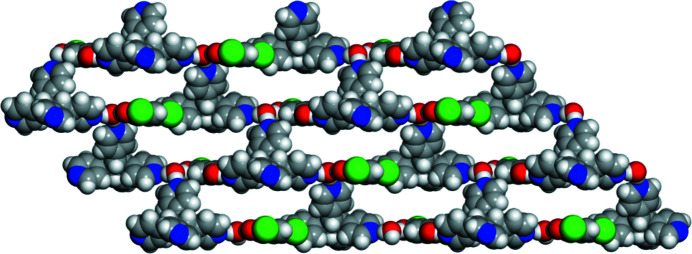
Extended view of the honeycomb network illustrating the observed channels.
